# Cholinesterase and Prolyl Oligopeptidase Inhibitory Activities of Alkaloids from *Argemone* *platyceras* (Papaveraceae)

**DOI:** 10.3390/molecules22071181

**Published:** 2017-07-14

**Authors:** Tomáš Siatka, Markéta Adamcová, Lubomír Opletal, Lucie Cahlíková, Daniel Jun, Martina Hrabinová, Jiří Kuneš, Jakub Chlebek

**Affiliations:** 1 Department of Pharmacognosy, Faculty of Pharmacy, Charles University, Akademika Heyrovského 1203, 500 05 Hradec Králové, Czech Republic; tomas.siatka@faf.cuni.cz; 2 Department of Pharmaceutical Botany and Ecology, ADINACO Research Group, Faculty of Pharmacy, Charles University, Akademika Heyrovského 1203, 500 05 Hradec Králové, Czech Republic; marketaadamcova@seznam.cz (M.A.); opletal@faf.cuni.cz (L.O.); cahlikova@faf.cuni.cz (L.C.); 3 Department of Toxicology and Military Pharmacy, Faculty of Military Health Sciences, University of Defence, Třebešská 1575, 500 01 Hradec Králové, Czech Republic; daniel.jun@unob.cz (D.J.); martina.hrabinova@unob.cz (M.H.); 4 Department of Inorganic and Organic Chemistry, Faculty of Pharmacy, Charles University, Akademika Heyrovského 1203, 500 05 Hradec Králové, Czech Republic; kunes@faf.cuni.cz

**Keywords:** *Argemone platyceras*, alkaloids, acetylcholinesterase, butyrylcholinesterase, prolyl oligopeptidase, Alzheimer’s disease

## Abstract

Alzheimer’s disease is an age-related, neurodegenerative disorder, characterized by cognitive impairment and restrictions in activities of daily living. This disease is the most common form of dementia with complex multifactorial pathological mechanisms. Many therapeutic approaches have been proposed. Among them, inhibition of acetylcholinesterase, butyrylcholinesterase, and prolyl oligopeptidase can be beneficial targets in the treatment of Alzheimer’s disease. Roots, along with aerial parts of *Argemone platyceras*, were extracted with ethanol and fractionated on an alumina column using light petrol, chloroform and ethanol. Subsequently, repeated preparative thin-layer chromatography led to the isolation of (+)-laudanosine, protopine, (–)-argemonine, allocryptopine, (–)-platycerine, (–)-munitagine, and (–)-norargemonine belonging to pavine, protopine and benzyltetrahydroisoquinoline structural types. Chemical structures of the isolated alkaloids were elucidated by optical rotation, spectroscopic and spectrometric analysis (NMR, MS), and comparison with literature data. (+)-Laudanosine was isolated from *A. platyceras* for the first time. Isolated compounds were tested for human blood acetylcholinesterase, human plasma butyrylcholinesterase and recombinant prolyl oligopeptidase inhibitory activity. The alkaloids inhibited the enzymes in a dose-dependent manner. The most active compound (–)-munitagine, a pavine alkaloid, inhibited both acetylcholinesterase and prolyl oligopeptidase with IC_50_ values of 62.3 ± 5.8 µM and 277.0 ± 31.3 µM, respectively.

## 1. Introduction

Alzheimer’s disease (AD) is an age-related, progressive, neurodegenerative disorder, with onset usually in later life. The disease is characterized by cognitive impairment, a variety of behavioral symptoms and restrictions in activities of daily living. AD is the most common form of dementia and the prevalence increases exponentially between the ages 65 to 85, doubling with every 5-year age group [1]. AD has two characteristic pathological hallmarks: extracellular accumulation of β-amyloid peptide (amyloid plaques), and intraneuronal formation of hyperphosphorylated τ-protein filaments (neurofibrillar tangles) leading to progressive loss of neurons and disintegration of the neural circuits, particularly in the cerebral cortex [2,3]. Use of the current available drugs in AD mostly relies on the cholinergic hypothesis supported by several observations that decreases in cholinergic transmission in the neocortex and hippocampus correlate with dementia severity [1]. Both acetylcholinesterase (AChE) and butyrylcholinesterase (BChE) are responsible for the breakdown of acetylcholine (ACh) in the synapses [4]. As AD progresses in severity, AChE concentrations and activity decrease and those of BChE increase [4,5,6]. In the normal brain, the relative proportions of cholinesterase activity are 99% for AChE and 1% for BChE; in advanced AD, the corresponding proportions are 65% and 35% (a change in ratio from 99:1 to 2:1) for AChE and BChE, respectively [5,7,8]. At advanced stages of this disorder, BChE may replace AChE in hydrolyzing brain ACh [5]. Therefore, inhibition of both enzymes can be beneficial to treat symptoms in AD [1]. Research focused on cholinesterase inhibitors of plant origin discovered that alkaloid extracts of some *Papaveraceae* species demonstrated interesting cholinesterase inhibitory activity [9,10,11,12].

Prolyl oligopeptidase (POP), also known as prolyl endopeptidase, represents another potential therapeutic hit in the treatment of AD. POP is an enzyme that cleaves peptides with a relatively small molecular weight at the carboxyl side of a proline residue, such as vasopressin, thyrotropin-releasing hormone, and substance P. POP is distributed in over 20 human tissue types with the highest activity found in skeletal muscles and the human brain, especially in the cortex. Previous studies suggested that POP could be related to neurodegeneration and disturbances in memory and cognition. Abnormal levels of POP activity have been found to be significantly higher in Alzheimer patients’ brains than in control patients [13]. The exact mechanism of memory and learning improving action of POP inhibitors is not yet fully explained and is still under study [14,15,16]. Inhibitors of POP may improve memory by blocking the metabolism of endogenous neuropeptides [13]. Neuropeptides such as vasopressin or thyrotropin-releasing hormone as well as substance P were described as cognition enhancers with positive modulatory effects on cerebral cholinergic activity. It has also been demonstrated that several neuropeptides can promote processes related to functional recovery following central nervous system damage. Deficiencies in substance P and vasopressin have been reported in postmortem studies of cerebral tissue derived from patients with neurodegenerative diseases [17]. A beneficial effect on mnemo-cognitive performance has been seen in patients with AD following thyrotropin-releasing hormone treatment [18,19]. Furthermore, POP might be involved in the processing of the C-terminal portion of the amyloid precursor protein, which has been revealed to injure neurons [20,21], suggesting that POP inhibitors may decrease the amyloid deposition [22]. However, a role of POP in the processing or degradation of β-amyloid appears to be unclear; POP could be associated with neuronal damage rather than β-amyloid accumulation [23]. Several in vivo experiments in animal models showed that POP inhibition led to neuroprotective, cognition-enhancing, and memory-enhancing effects; short-term, long-term, reference and working memory were positively influenced [14]. Some clinical trials with POP inhibitors in the treatment of cognitive deficits have been performed. The most reported compound was S-17092. It showed cognition-enhancing properties in healthy elderly subjects and could improve a delayed verbal memory task [24]. In addition, some mood-stabilizing potential in healthy young volunteers has been observed [25]. Thus, POP-inhibiting compounds appear as a promising therapeutic approach to the treatment of AD. POP inhibitors were also found among natural products of different structural types (e.g., phenolics, benzofurans, terpenes, peptides, and alkaloids) [13,26,27,28,29].

*Argemone platyceras* Link et Otto, a species of prickly poppy, belonging to the family Papaveraceae, is a spiny annual herb with incised leaves and white flowers [30]. The plant is widely distributed throughout Mexico and is commonly known as “chicalota”, which is used in the form of flower infusion by several Mexican ethnic groups as a remedy for cough, bronchitis and pneumonia [31]. Two groups of secondary metabolites are documented for *A. platyceras*: isoquinoline alkaloids and flavonoids (isoquercitrin and rutin) [31]. Many tertiary and quaternary isoquinoline alkaloids have been found in *A. platyceras* (Table 1), the major ones being (–)-platycerine, (–)-munitagine, (–)-argemonine and protopine in the overground parts [32,33,34,35], and protopine in the roots [33].

No systematic studies have been reported regarding the pharmacological properties of *A*. *platyceras*. Methanol extracts of the leaves and flowers showed anti-asthmatic activity in an allergic asthma model (isolated guinea pig trachea), and bioassay-guided fractionation led to isoquercitrin being found as the active principle responsible for it [31].

This study deals with the isolation of tertiary alkaloids from the aerial parts and roots of *A. platyceras* (quaternary alkaloids might have problems to cross the blood-brain barrier [39]), and their ability to inhibit erythrocyte AChE and plasma BChE from human blood. Additionally, these compounds were also tested for their inhibitory activity on recombinant POP, since it has been found that some isoquinoline alkaloids possess a POP inhibitory effect [27].

## 2. Results and Discussion

### 2.1. Choice of Plant Material

During a screening focused on cholinesterase inhibitors of plant origins, we found a promising cholinesterase inhibitory activity of an ethyl-acetate alkaloid extract of *A. platyceras* aerial parts and roots with IC_50_ values for AChE of 15.7 ± 2.9 µg/mL and IC_50_ BChE of 25.5 ± 1.8 µg/mL. Galanthamine, huperzine A and eserine were used as positive controls.

### 2.2. Isolated Alkaloids

**Figure 1:**
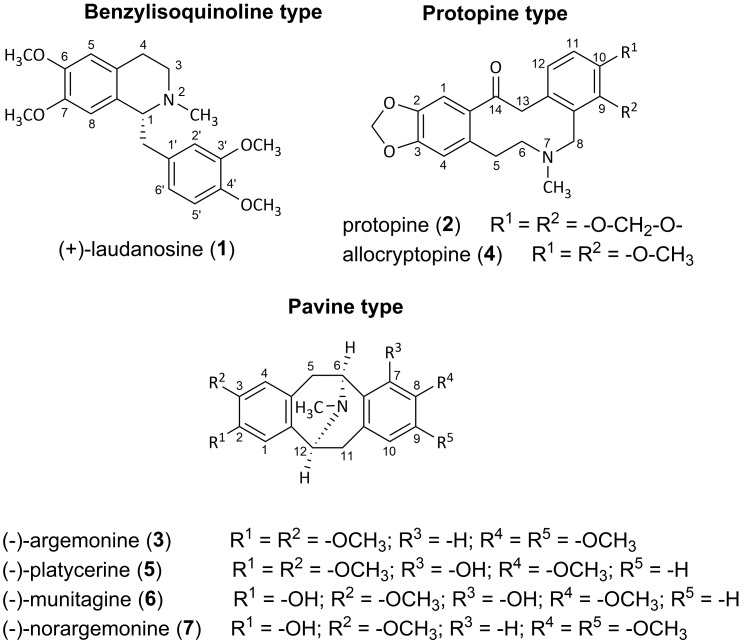
Tertiary alkaloids isolated from *A. platyceras* in our study.

Column chromatography (CC) and subsequent preparative thin-layer chromatography (TLC) led to the isolation of seven compounds (**1**–**7**) belonging to pavine, protopine and benzylisoquinoline structural types. The chemical structures of the isolated alkaloids were elucidated by means of optical rotation, spectroscopic (^1^H- and ^13^C-NMR) and spectrometric (GC-MS, ESI) analyses and by comparison of the obtained data with those in the literature [17,18,19,20,21,22,23,24,25,26,27,28,29,30,31,32,33,34,35,36,38,40,41,42,43,44,45,46,47]. The compounds were determined as (+)-laudanosine (**1**), protopine (**2**), (–)-argemonine (**3**), allocryptopine (**4**), (–)-platycerine (**5**), (–)-munitagine (**6**) and (–)-norargemonine (**7**) (Figure 1).

This study is the first report about the isolation of (+)-laudanosine (**1**), an opium alkaloid, from *A*. *platyceras*. In the genus *Argemone* compound **1** has been identified as a minor alkaloid in *A*. *grandiflora* only [48]. Laudanosine, arising from the key branch point intermediate (+)-reticuline, as other benzylisoquinoline alkaloids, belongs to the benzyltetrahydroisoquinolines [49]. The remaining alkaloids (**2**–**7**) have been previously isolated from *A. platyceras* (Table 1). The protopine alkaloids protopine (**2**) and allocryptopine (**4**) are very common in species of Papaveraceae, Fumariaceae, Berberidaceae, Rutaceae, Ranunculaceae, and Sapindaceae. Protopines possess, as free tricyclic bases, a ten-membered heterocyclic ring containing one tertiary nitrogen and carbonyl group, and, under acidic conditions, they form tetracyclic salts [50]. (–)-Argemonine (**3**), (–)-platycerine (**5**), (–)-munitagine (**6**) and (–)-norargemonine (**7**) belong to a small group of alkaloids called pavines, which are all derived from benzylisoquinolines functionalized in the ring B. Pavines are found in four plant families, namely Papaveraceae, Berberidaceae, Lauraceae, and Ranunculaceae. Within the Papaveraceae, pavines are known to occur in the genera *Argemone* and *Eschscholtzia* [51].

### 2.3. Cholinesterase Inhibitory Activity of Isolated Alkaloids

NMDA blockers and cholinesterase inhibitors (ChEIs) are used in AD therapy [52,53,54] and are being tested in clinical trials [53,54]. The spectrum of therapeutically used drugs is limited; the U.S. Food and Drug Administration (FDA) has approved donepezil, galanthamine and rivastigmine as ChEIs to treat the symptoms of mild to moderate AD. All these drugs inhibit both enzymes, but with different potency. From the pharmacological point of view, galanthamine and donepezil are taken as selective AChE inhibitors, while rivastigmine is a dual inhibitor of cholinesterases [52,55,56]. As mentioned above, both AChE and BChE are responsible for the breakdown of ACh in the synapses. Thus, inhibition of both enzymes represents a beneficial approach in AD treatment.

**Figure 2:**
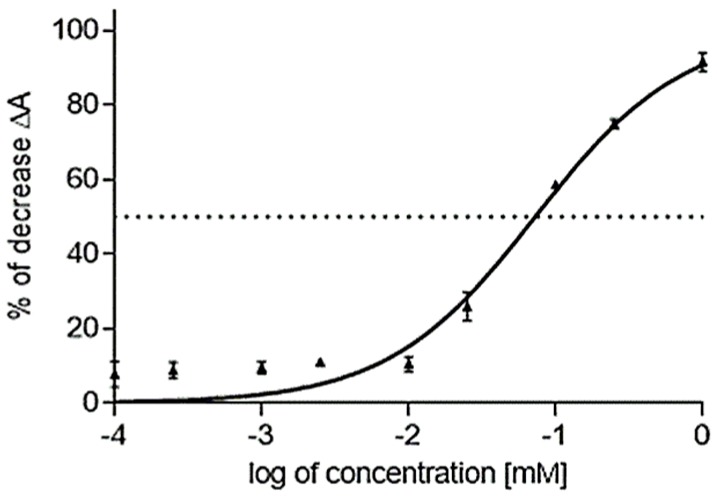
The dose-response curve of (–)-munitagine (IC_50_ calculation of its AChE inhibitory activity).

All isolated compounds were tested for cholinesterase inhibitory activity using Ellman’s method [57]. Red blood cell lysate was used as a source of AChE and human plasma as a source of BChE. The results are expressed as IC_50_ values, with galanthamine, huperzine A and eserine as positive controls (Table 2). The isolated tertiary isoquinoline alkaloids showed weaker cholinesterase inhibition than the standards. (–)-Munitagine (**6**) was the most potent compound inhibiting AChE in a dose-dependent manner with an IC_50_ = 62.3 ± 5.8 µM (Figure 2). Moreover, **6** was found to be a selective inhibitor of AChE: towards BChE it was considered almost inactive (IC_50_ = 837.4 ± 23.0 µM). Pavines, according to our obtained results (**3**, **5**–**7**) and those reported in the literature [58], possess either weak or no activity on BChE (IC_50_ > 837.4 µM), but generally show mild AChE inhibitory activity, excluding the moderate AChE inhibitors (–)-munitagine, (–)-caryachine and (–)-californidine with IC_50_ values of 62.3 ± 5.8 µM, 19.6 ± 0.4 µM [58] and 36.7 ± 0.9 µM [58], respectively.

Furthermore, in our work, the protopine alkaloids, protopine (**2**) and allocryptopine (**4**), demonstrated mild cholinesterase inhibition (protopine: IC_50_ AChE = 230.0 ± 21.0 µM; IC_50_ BChE = 208.9 ± 17.7 µM), and allocryptopine IC_50_ AChE = 250.0 ± 25.0 µM; IC_50_ BChE = 530.0 ± 28.2 µM). Contrary to our results, in the review of Sener and Orhan, it is mentioned that protopine and allocryptopine, isolated as active substances from *Fumaria vaillantii*, were identified as potent AChE inhibitors with IC_50_ values of 1.8 and 1.3 µM, respectively [59]. The contrasting results for AChE inhibitory activity of protopine and allocryptopine could be explained by the use of different conditions in the assays e.g., the source of enzyme (AChE from electric eel or human AChE), concentration of substrate (acetylthiocholine)–protopine was found to be a dose-dependent, specific, reversible and competitive AChE inhibitor [50]. Thus, it is very difficult to compare the obtained data with those in the literature, and for the explanation of the cholinesterase activity of these compounds, further biological studies are needed.

Finally, (+)-laudanosine (**1**), a benzyltetrahydroisoquinoline with four methoxy substituents on the skeleton, was considered inactive on cholinesterases, with IC_50_ values for AChE and BChE > 1000 µM. This compound has not been tested for its ability to inhibit cholinesterase so far. In comparison to the cholinesterase inhibitory activity of benzylisoquinolines in the literature to our found results of **1**, it seems that cholinesterase inhibitory activity of this structural type is connected to the presence of one hydroxyl (OH) group on the benzene ring at positions 3′ or 4′ in the case of the 6-hydroxy-7-methoxybenzylisoquinolines. For instance, (+)-reticuline and (+)-*N*-methylcoclaurine, which are selective BChE inhibitors with IC_50_ values of 33.6 ± 3.0 and 15.0 ± 1.4 µM (IC_50_ values for AChE inhibition were >220 µM) [60]. 6,7-Methyldioxybenzylisoquinoline with OH and methoxy groups at the 3′ and 4′ positions on the benzene ring, respectively, showed similar anti-BChE activity to reticuline (IC_50_ BChE = 28 µM; IC_50_ AChE = 102 µM) [58]. Furthermore, the 6,7-methyldioxy substituent on the benzylisoquinoline skeleton seems to be responsible for better AChE inhibition. The above mentioned compound and (+)-canadaline inhibited AChE, with canadaline giving an IC_50_ value of 20.1 µM [61]. The study of Markmee et al. has provided more insights into the SAR of 1-benzylisoquinoline derivatives on AChE inhibition [62]. Additional studies are underway to determine structural requirements of this class of benzylisoquinolines. Considering that the EtOAc alkaloid extract of *A*. *platyceras* demonstrated promising cholinesterase inhibitory activity with values for IC_50_ AChE of 15.7 ± 2.9 µg/mL and IC_50_ BChE of 25.5 ± 1.8 µg/mL. It seems that the resultant activity was not caused by a synergistic effect of the isolated compounds [63], but probably due to the presence of quaternary alkaloids in the extract (chelerythrine, berberine, coptisine, and sanguinarine are very potent inhibitors of cholinesterases) [64,65,66,67], and they were found in this plant (Table 1). Additionally, most of these quaternary alkaloids are soluble in some lipophilic solvents [68,69].

### 2.4. Prolyl Oligopeptidase Inhibitory Activity of Isolated Alkaloids

Similarly, POP has been suggested to participate in the pathogenesis of AD, and, therefore, POP inhibition can become another supporting therapeutic approach in AD treatment. Consequently, given the complexity and interconnected pathological pathways of the disease, the research focused on compounds influencing more therapeutic targets (a multi-target drug approach) involved in AD is needed. Thus, finding of inhibitors with anti-cholinesterase and POP inhibitory activity maybe provide a more effective treatment of AD.

**Figure 3:**
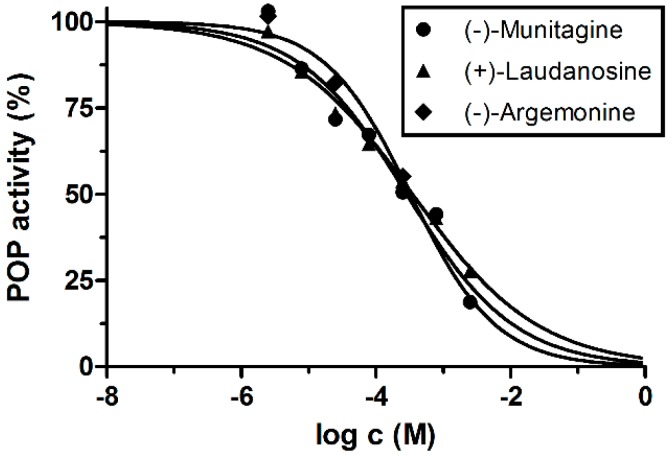
The dose-response curves of the most potent POP inhibitors (–)-munitagine, (+)-laudanosine and (–)-argemonine (calculation of their IC_50_ values).

Compounds isolated in sufficient amounts (**1**–**6**) were tested for their ability to inhibit POP and our data correspond with those in the literature [27]. *Z*-Pro-prolinal (a synthetic compound) and berberine were used as positive standards with IC_50_ values of 3.27 ± 0.02 nM and 142.0 ± 21.5 µM, respectively. None of the isolated alkaloids demonstrated a similar potent inhibition as the reference compounds. The most active of the isolated compounds were (–)-munitagine (**6**), (–)-argemonine (**3**) and (+)-laudanosine (**1**), with IC_50_ values of 277.0 ± 31.3 µM, 337.0 ± 83.1 µM and 341.0 ± 37.5 µM, respectively (Figure 3). (–)-Platycerine (**3**), isolated and tested for POP inhibition previously [27], was a twofold weaker POP inhibitor than (–)-argemonine; its IC_50_ was 687.0 µM. Protopine (**2**) and allocryptopine (**4**) were considered inactive (IC_50_ > 1000 µM). It seems that benzylisoquinoline and pavine types possess anti-POP activity, reversely; protopines lack POP activity.

## 3. Experimental

### 3.1. Plant Material

Seeds of *A. platyceras* for the plant cultivation were obtained from the Centre of Medicinal Plants of Faculty of Medicine, Masaryk University in Brno, the Czech Republic, in 2012. Plants were cultivated in the Botanical Garden of Medicinal Plants of the Faculty of Pharmacy in Hradec Králové, Charles University, the Czech Republic in 2013; the collection of the plants was in the same year. Prof. Dr. L. Opletal performed botanical identification and verification. A voucher specimen has been deposited in the herbarium of the Faculty of Pharmacy in Hradec Králové.

### 3.2. General Experimental Procedures

^1^H-NMR (500 MHz) and ^13^C-NMR (125.7 MHz) spectra were recorded on a VNMR S500 NMR spectrometer (Varian, Palo Alto, CA, USA). The ESI-MS (electrospray ionization mass spectrometry) analysis was performed on a Thermo Finnigan LCQDuo spectrometer and EI-MS on an Agilent 7890A GC 5975 inert MSD operating in EI mode at 70 eV (Agilent Technologies, Santa Clara, CA, USA). A DB-5 column (30 m × 0.25 mm × 0.25 μm, Agilent Technologies, USA) was used. The temperature program was set at 100–180 °C at 15 °C/min, 1 min hold at 180 °C and 180–300 °C at 5 °C/min and 5 min hold at 300 °C, detection range *m*/*z* 40–600. The injector temperature was 280 °C. The flow-rate of carrier gas (helium) was 0.8 mL/min. A split ratio of 1:10 was used. Optical rotation was measured on an P3000 polarimeter (A. Krüss Optronic, Germany) in chloroform (CHCl_3_). TLC was carried out on Merck pre-coated silica gel 60 F254 plates and for visualization of alkaloids on the TLC plates UV detection (254 and 366 nm) and spraying with Dragendorff´s reagent were performed. As solvents for mobile phases of TLC were used diethylamine (Et_2_NH), cyclohexane (C_6_H_12_), toluene (C_6_H_5_CH_3_), ethyl-acetate (EtOAc), acetonitrile (ACN), acetone (DMK) and aqueous ammonia solution (NH_4_OH) (Penta, Ing. Švec, Praha, Czech Republic), and trifluoroacetic acid (TFAA, Sigma Aldrich). Neutral aluminum oxide (Al_2_O_3_; 50–200 µm; Lach-Ner, Neratovice, Czech Republic) was used for CC.

### 3.3. Extraction and Isolation of Alkaloids

Dried and powdered aerial parts, along with roots, of *A. platyceras* (7.95 kg; 7.52 kg and 0.43 kg, respectively) were extracted with EtOH (96%, 2×) at room temperature for 48 h. The combined macerate was filtered and evaporated to dryness and a dark-greenish viscous crude extract (190 g) was obtained. To the extract, 2 L H_2_O was added, the suspension was heated to 70 °C and acidified with 2% HCl to pH~1, filtered, and the filtrate was alkalized with 10% Na_2_CO_3_ to pH 9–10 and tertiary alkaloids were exhaustively extracted with CHCl_3_ (3 × 700 mL). The remaining quaternary alkaloids in the water phase were not further processed. The organic layer was evaporated to give 19 g of a dark-brownish viscous alkaloid concentrate, which was subsequently purified by dissolving in 2% HCl, filtered, extracted with diethyl ether (Et_2_O; 2 × 200 mL) to remove non-alkaloid compounds, alkalized with 10% Na_2_CO_3_ and extracted with Et_2_O (4 × 300 mL). The Et_2_O layer was treated with 2% HCl (4 × 150 mL), subsequently alkalized with Na_2_CO_3_, and the water layer extracted with Et_2_O (3 × 300 mL). The combined Et_2_O extract was evaporated to give 10.35 g of the brownish viscous extract. This was further fractionated by CC on neutral Al_2_O_3_ eluting with light petrol + CHCl_3_ (4:1, 3:1, 7:3, 3:2, 1:1, 1:3), CHCl_3_ and CHCl_3_ + EtOH (1:1). Fractions (250 mL) were collected and monitored by TLC leading to 144 fractions that were combined into 8 (A–H) fractions. Preparative TLC (C_6_H_12_ + Et_2_NH, 9:1, 2×) of fraction A (220 mg) led to the isolation of amorphous white compound **1** (18 mg). Crystallization of fraction C (1.68 g) from CHCl_3_ + EtOH (1:1) led to compound **2** (900 mg). The parent residue (720 mg) was subjected to preparative TLC (C_6_H_12_ + C_6_H_5_CH_3_ + Et_2_NH, 45:45:10, 2×) to give 2 sub-fractions C1 (20 mg), and C2 (90 mg) from which white crystalline compound **3** (27.6 mg) was obtained by crystallization from 25% aqueous EtOH. Crystallization of fraction E (white powder, 66 mg) from CHCl_3_ + EtOH (1:1) led to compound **4** (32 mg). Fraction F (3.24 g) was treated with 2% HCl, then alkalized and extracted with Et_2_O. Subsequent repeated crystallization from Et_2_O led to the isolation of compound **5** (white powder, 500 mg; [11]). Fraction G (762 mg) was treated by preparative TLC (EtOAC + ACN + TFAA, 40:5:0.1, 4×) resulting in 2 sub-fractions G1-2. Repeated crystallization of G1 (200 mg) gave compound **6** as a white crystalline powder (31.5 mg). Sub-fraction G2 (159 mg) was further chromatographed by preparative TLC (C_6_H_12_ + DMK + NH_4_OH, 40:60:1, 1×) to give compound **7** (7.1 mg). Based on GC-MS and TLC analysis, fractions B (650 mg), D (230 mg) and H (353 mg) were not further separated to give pure compounds (presence of compounds previously found in the above-mentioned fractions C and E; fraction H due to trace amount of detected alkaloids).

### 3.4. Characterization Data

*(+)-Laudanosine* (**1**): amorphous white solid; ${\left[\alpha \right]}_{D}^{25}$ = +34° (c 1.96; CHCl_3_); EI-MS (*m*/*z*) 357 (<1), 206 (100), 190 (12); ESI-MS *m*/*z* [M + H]^+^ 358 (100), 218 (10), 206 (6); ^1^H-NMR (500 MHz, CDCl_3_) δ 6.78 (1H, d, *J* = 8.1 Hz, H5´), 6.65 (1H, dd, *J* = 8.1 Hz, *J* = 2.1 Hz, H6´), 6.62 (1H, d, *J* = 2.1 Hz, H2´), 6.57 (1H, s, H5), 6.07 (1H, s, H8), 3.86 (3H, s, OCH_3_), 3.85 (3H, s, OCH_3_), 3.80 (3H, s, OCH_3_), 3.74–3.69 (1H, m, H1), 3.59 (3H, s, OCH_3_), 3.23–3,13 (2H, m, H3, H1´´), 2.89–2.75 (3H, m, H3, H4, H1´´), 2.65–2.57 (1H, m, H4), 2.56 (3H, s, NCH_3_); ^13^C-NMR (125 MHz, CDCl_3_) δ 148.6, 147.4, 147.3, 146.3, 132.4 129.7, 125.9, 121.9, 113.0, 111.2, 111.1, 111.0, 64.9, 55.9, 55.8, 55.8, 55.6, 46.9, 42.6, 40.9, 25.4.

*Protopine* (**2**): white crystals; m.p. 206–207 °C; EI-MS *m*/*z* 353 (5), 190 (10), 163 (25), 148 (100); ^1^H-NMR (500 MHz, CDCl_3_) δ 6.90 (1H, s, H1), 6.68 (1H, d, *J* = 8.0 Hz, H11), 6.65 (1H, d, *J* = 8.0 Hz, H12), 6.63 (1H, s, H4), 5.94 (2H, s, H15), 5.91 (2H, s, H16), 3.96–3.69 (2H, m, H13), 3.69–3.45 (2H, m, H8), 3.23–2.84 (1H, m, H5), 2.76–2.40 (3H, m, H5, H6, H6), 1.95 (3H, s, NCH_3_); ^13^C-NMR (125 MHz, CDCl_3_) δ 192.4 (C14), 148.0 (C3), 146.2 (C9), 146.0 (C10), 145.9 (C2), 135.8 (C14a), 132.5 (C4a), 128.8 (C12a), 124.9 (C12), 117.6 (C8a), 110.4 (C4), 108.0 (C1), 106.7 (C11), 101.2 (C15), 100.8 (C16), 57.7 (C6), 50.9 (C8), 46.2 (C13), 41.5 (NCH_3_), 31.5 (C5).

*(–)-Argemonine* (**3**): white crystals; m.p. 152–153 °C; ${\left[\alpha \right]}_{D}^{25}$ = –202° (c 0.51; CHCl_3_); EI-MS (*m*/*z*) 355 (30), 354 (20), 204 (100); ^1^H-NMR (500 MHz, CDCl_3_) δ 6.62 (2H, s, H4, H10), 6.46 (2H, s, H1, H7), 4.01 (2H, d, *J* = 5.9 Hz, H5, H11), 3.86 (6H, s, OCH_3_), 3.79 (6H, s, OCH_3_), 3.41 (2H, dd, *J* = 16.1 Hz, *J* = 5.9 Hz, H6, H12), 2.60 (2H, d, *J* = 16.1 Hz, H6, H12), 2.54 (3H, s, NCH_3_); ^13^C-NMR (125 MHz, CDCl_3_) δ 147.8, 147.5, 130.0, 123.9, 111.5, 110.0, 56.4, 55.9, 55.7, 40.9, 33.6.

*Allocryptopine* (**4**): white crystals; m.p. 159–161 °C; EI-MS (*m*/*z*) 369 (5), 354 (5), 283 (15),206 (20), 164 (100), 162 (25), 149 (30), 134 (25); ^1^H-NMR (500 MHz, CDCl_3_) δ 6.95 (1H, s); 6.91 (1H, d); 6.80 (1H, d, *J* = 8.24 Hz); 6.63 (1H, s); 5.94 (2H, s); 3.86 (3H, s); 3.78 (3H, s); 3.72 (2H, bs); 2.2–3.5 (3H, m); 1.86 (3H, s); ^13^C-NMR (125 MHz, CDCl_3_) δ 193.6, 151.8, 148.3, 147.9, 146.6, 136.3, 133.1, 129.8, 128.8, 128.0, 110.8, 110.7, 109.5, 101.4, 61.0, 57.8, 55.9, 50.4, 46.5, 41.4, 32.6.

*(–)-Platycerine* (**5**): white crystals; m.p. 130–131 °C; ${\left[\alpha \right]}_{D}^{25}$ = −264° (c 0.43; CHCl_3_); EI-MS (*m*/*z*) 341(42), 340 (25), 204 (100), 190 (28); ^1^H-NMR (500 MHz, CDCl_3_) δ 6.67 (1H, d, *J* = 8.3 Hz, H2), 6.60 (1H, s, H10), 6.52 (1H, d, *J* = 8.3 Hz, H1), 6.46 (1H, s, H7), 5.97 (1H, bs, OH), 4.46 (1H, d, *J* = 5.7 Hz, H5), 4.09 (1H, d, *J* = 5.7 Hz, H11), 3.85 (3H, s, OCH_3_), 3.82 (3H, s, OCH_3_), 3.77 (3H, s, OCH3), 3.48 (1H, dd, *J* = 16.5 Hz, *J* = 5.7 Hz, H12), 3.40 (1H, dd, *J* = 16.5 Hz, *J* = 5.7 Hz, H6), 2.80 (1H, d *J* = 16.5 Hz, H6), 2.69 (1H, d, *J* = 16.5 Hz, H12), 2.61 (3H, s, NCH_3_); ^13^C-NMR (125 MHz, CDCl_3_) δ 147.9, 147.5, 144.3, 141.9, 128.6, 124.6, 124.4, 123.5, 119.6, 111.5, 109.9, 109.5, 56.4, 56.0, 55.9, 55.7, 51.8, 40.6, 33.0, 30.7.

*(–)-Munitagine* (**6**): white crystals; m.p. 168–169 °C; ${\left[\alpha \right]}_{D}^{25}$ = −178° (c 0.26; CHCl_3_); EI-MS (*m*/*z*) 327 (30), 326 (20), 190 (100); ^1^H-NMR (500 MHz, CDCl_3_) δ 6.66 (1H, d, *J* = 8.2 Hz, H2), 6.66 (1H, s, H10), 6.50 (1H, d, *J* = 8.2 Hz, H1), 6.44 (1H, s, H7), 5.72 (1H, bs, OH), 4.38 (1H, d, *J* = 5.7 Hz, H5), 3.99 (1H, d, *J* = 5.7 Hz, H11), 3.83 (3H, s, OCH_3_), 3.78 (3H, s, OCH_3_), 3.43–3.31 (2H, m, H6, H12), 2.75 (1H, d *J* = 16.1 Hz, H6), 2.62 (1H, d, *J* = 16.1 Hz, H12), 2.55 (3H, s, NCH_3_); ^13^C-NMR (125 MHz, CDCl_3_) δ 144.4, 144.1, 143.8, 141.8, 130.4, 125.5, 124.3, 124.3, 119.7, 112.7, 110.9, 109.2, 56.1, 56.0, 55.7, 51.6, 40.8, 32.9, 31.0.

*(–)-Norargemonine* (**7**): white crystals; m.p. 237−238 °C; ${\left[\alpha \right]}_{D}^{25}$ = −152° (c 0.09; CHCl_3_); EI-MS (*m*/*z*) 341 (60), 340 (35), 204 (95), 190 (100); ^1^H-NMR (500 MHz, CD_3_OD) δ 6.76 (1H, s, H4), 6.73 (1H, s, H7), 6.56 (1H, s, H1), 6.41 (1H, s, H10), 4.04–4.00 (2H, m, H5, H11), 3.83 (3H, s, OCH_3_), 3.81 (3H, s, OCH_3_), 3.74 (3H, s, OCH_3_), 3.42–3.35 (2H, m, H6, H12), 2.68–2.53 (2H, m, H6, H12), 2.49 (3H, s, NCH_3_); ^13^C-NMR (125 MHz, CD_3_OD) δ 149.5, 149.2, 147.9, 146.5, 131.1, 129.8, 125.3, 125.2, 116.0, 113.2, 111.9, 111.5, 57.6, 57.6, 56.5, 56.4, 56.4, 40.7, 34.6, 34.3.

Purity of isolated compounds was ≥95% (by NMR and GC).

### 3.5. Biological Assays

#### 3.5.1. Materials

Acetylthiocholine iodide (ATChI), butyrylthiocholine iodide (BTChI), berberine chloride (>95%), recombinant POP and its substrate, *Z*-Gly-Pro-*p*-nitroanilide (≥99%) and eserine (≥99%) were purchased from Sigma-Aldrich (Prague, Czech Republic); galanthamine hydrobromide (>98%) from Changsha Organic Herb Inc. (Changsha City, China), and huperzine A (98%) from Tai´an zhonghui Plant Biochemical Co., Ltd. (Xintai, China). Red blood cell lysate was used as a source of AChE, and human plasma as a source of BChE.

#### 3.5.2. Preparation of Enzymes for AChE, BChE Assays

Enzymes were prepared from freshly drawn blood (taken from healthy volunteers), to which 2 mL 3.4% sodium citrate (*w*/*v*) per 18 mL blood was added, according to Steck and Kant [70], with slight modification. Briefly, plasma (BChE) was removed from the whole blood by centrifugation at 4000 rpm in a Boeco U-32R centrifuge fitted with a Hettich 1611 rotor. Red blood cells were transferred to 50 mL tubes and washed three times with 5 mM phosphate buffer (pH 7.4) containing 150 mM sodium chloride (centrifugation under same conditions). The washed erythrocytes were stirred with 5 mM phosphate buffer (pH 7.4) for 10 min to ensure lysis. The lysed cells were dispensed for subsequent measurement. Activity of each enzyme preparation was measured immediately after preparation and adjusted with 5 mM phosphate buffer (pH 7.4) to reach activity of blank sample A = 0.08–0.15 for AChE and A = 0.15–0.20 for BChE [71].

#### 3.5.3. Cholinesterases Assays

AChE and BChE activities were determined using a modified Ellman’s method with ATChI and BTChI as substrates, respectively [36]. Briefly, 8.3 μL of either blood cell lysate or plasma dilutions (at least six different concentrations), 283 μL of 5 mM 5,5′-dithiobis-2-nitrobenzoic acid (DTNB) and 8.3 μL of either the sample dilution in dimethyl sulfoxide (DMSO) (40 mM, 10 mM, 4 mM, 1 mM, 0.4 mM and 0 mM) or the blank sample (DMSO) were added into wells of a polystyrene 96-well microplate with a flat and clear bottom. The reaction was initiated by addition of 33.3 μL 10 mM substrate (ATChI or BTChI). The final proportion of DTNB and substrate was 1:1. The increase of absorbance (ΔA) at 436 nm for AChE and 412 nm for BChE was measured for 1 min at 37 °C using a spectrophotometer (SynergyTM HT Multi-Detection Microplate Reader). Each measurement was repeated six times for every concentration of enzyme preparation. The % inhibition was calculated according to the formula:

$$\%I=100-\left(100\times \frac{\Delta A_{\mathrm{Bl}}}{\Delta A_{\mathrm{Sa}}}\right)$$

where ΔA_Bl_ is the increase of absorbance of the blank sample and ΔA_Sa_ is the increase of absorbance of the measured sample. Inhibition potency of the tested compounds was expressed as an IC_50_ value (the concentration of an inhibitor, which causes 50% cholinesterase inhibition) [50].

#### 3.5.4. Prolyl Oligopeptidase Assay

Prolyl oligopeptidase (POP; EC 3.4.21.26) was dissolved in phosphate buffered saline (PBS; 0.01 M Na/K phosphate buffer, pH 7.4, containing 137 mM NaCl and 2.7 mM KCl); the specific activity of the enzyme was 0.2 U/mL. The assay was performed in standard polystyrene 96-well microplates with a flat and clear bottom. Stock solutions of tested compounds were prepared in dimethyl sulfoxide (DMSO; 10 mM). Dilutions (10^−3^–10^−7^ M) were prepared from the stock solution with deionized H_2_O; the control was performed with the same DMSO concentration. POP substrate, (*Z*)-Gly-Pro-*p*-nitroanilide, was dissolved in 50% 1,4-dioxane (5 mM). For each reaction, PBS (170 µL), tested compound (5 µL), and POP (5 µL) were incubated for 5 min at 37 °C. Then, substrate (20 µL) was added and the microplate was incubated for 30 min at 37 °C. The formation of *p*-nitroanilide, directly proportional to the POP activity, was measured spectrophotometrically at 405 nm using a microplate ELISA reader (Multi-mode microplate reader Synergy 2, BioTek Instruments Inc., Winooski, VT, USA). Inhibition potency of tested compounds was expressed as an IC_50_ value [27].

#### 3.5.5. Statistical Analysis

Calculations were performed using Microsoft Excel software (Redmont, WA, USA) and GraphPad Prism version 6.07 for Windows (GraphPad Software, San Diego, CA, USA).

## 4. Conclusions

This work focused on isolation of the tertiary alkaloids of *A. platyceras* and determination of their acetylcholinesterase, butyrylcholinesterase and prolyl oligopeptidase inhibitory activities. Seven alkaloids were isolated in sufficient amounts. (+)-Laudanosine, was isolated from *A. platyceras* for the first time. The alkaloids showed a dose-dependent inhibition in the enzyme assays. Some of them were tested for these biological effects for the first time. One of them, munitagine, is a pavine alkaloid, which was the most active compound. Moreover, considering the multifactorial pathological mechanisms of Alzheimer’s disease, an interesting finding is the dual activity of munitagine hitting two disorder targets–it inhibits acetylcholinesterase and prolyl oligopeptidase.

## Figures and Tables

**Table 1:** Isoquinoline alkaloids isolated from *A. platyceras* Link et Otto so far.

Alkaloid	Plant Part [Literature]
(–)-platycerine	aerial parts [33,34,35], roots [33]
(–)-munitagine	aerial parts [35], aerial parts + roots [36] *
(–)-argemonine	aerial parts [33,34,35], aerial parts + roots [36] *
protopine	aerial parts [32,33,34,35], roots [33]
(+)-reticuline	aerial parts [35], aerial parts + roots [36] *
(–)-norargemonine	aerial parts [32,33,34], roots [33]
allocryptopine	aerial parts [32,34,35], roots [33]
chelerythrine	aerial parts [34]
coptisine	aerial parts [33,34], roots [33,37]
berberine	aerial parts [33,34,35], roots [33,37]
(–)-*O*-methylplatycerine	aerial parts [35]
corysamine	aerial parts [34], roots [37]
sanguinarine	aerial parts [33,34,35], roots [33]
(–)-scoulerine	aerial parts + roots [36] *
(–)-platycerine methohydroxide	aerial parts [34], roots [37]
(–)-argemonine methohydroxide	aerial parts [34]
(–)-α-stylopine methohydroxide	aerial parts [34,35], roots [37]
(+)-magnoflorine	aerial parts [35], roots [37]
(–)-α-canadine methohydroxide	roots [37]
(–)-cyclanoline	roots [37]
armepavine	aerial parts [38] **
escholtzine	aerial parts [38] **
isonorargemonine	aerial parts [38] **
bisnorargemonine	aerial parts [38] **
cryptocavine	aerial parts [38] **
cryptopalmatine	aerial parts [38] **

* Alkaloids isolated from 90% aerial parts + 10% roots; ** alkaloids identified by means of GC-MS in a plant extract, but not isolated.

**Table 2:** *A. platyceras* alkaloids and their acetylcholinesterase (AChE), butyrylcholinesterase (BChE), and prolyl oligopeptidase (POP) inhibitory activity.

Compounds	AChE (IC_50_, µM) ^a^	BChE (IC_50_, µM) ^a^	POP (IC_50_, µM) ^a^
**1**	>1000	>1000	341.0 ± 37.5
**2**	230.0 ± 21.0	208.9 ± 17.7	>1000
**3**	>1000	885.5 ± 119.5	337.0 ± 83.1
**4**	250.0 ± 25.0	530.0 ± 28.2	>1000
**5**	223.7 ± 19.6	>1000	687.0 ± 74.0 ^b^
**6**	62.3 ± 5.8	837.4 ± 23.0	277.0 ± 31.3
**7**	205.2 ± 11.7	>1000	n.d ^c^
Galanthamine *	1.71 ± 0.07	42.30 ± 1.3	n.d.
Huperzine A	0.033 ± 0.001	>1000	n.d.
Serine *	0.063 ± 0.001	0.13 ± 0.004	n.d.
Berberine *	n.d.	n.d.	142.0 ± 21.5
*Z*-Pro-prolinal *	n.d	n.d	3.27 ± 0.02 × 10^−3^

^a^ Results are the means ± S.E.M. of three independent replications; ^b^ reference [27]; ^c^ insufficient amounts for assay measurement; * standard; n.d. not determined.
